# Prostate cancer diagnosis and management: current practices in Africa a consultant-based survey

**DOI:** 10.3389/fruro.2025.1496951

**Published:** 2025-03-17

**Authors:** Marcella Derboise Christelle Biyouma, Kaleab Habtemichael Gebreselassie, Saleh Abdelkerim Nedjim, Ouima Justin Dieudonné Ziba, Axel Stéphane Nwaha Makon, Anteneh Tadesse Kifle, Taofiq Olayinka Mohammed, Ayun Kotokai Cassell, Bencherki Youssef, Sissoko Idrissa, Orgeness Jasper Mbwambo, Mahamat Ali Mahamat, Rachid Aboutaieb, Tagang Titus Ngwa‐Ebogo, Alain Khassim Ndoye, Emiola Oluwabunmi Olapade-Olaopa, Fru Fobuzshi Angwafo

**Affiliations:** ^1^ Modern Urology For Africa (MUFA), Casablanca, Morocco; ^2^ Urology Unit, Department of Surgery, Douala Laquintinie Hospital, Douala, Cameroon; ^3^ Urology Unit, Worabe Comprehensive Specialized Hospital, Worabe, Ethiopia; ^4^ Urology Unit, Department of Surgery, Hôpital Universitaire La Renaissance, N’djamena, Chad; ^5^ Urology and Andrology Unit, Department of Surgery and Subspecialties, Yalgado Ouédraogo Teaching Hospital, Ouagadougou, Burkina Faso; ^6^ Department of Surgery and Subspecialty, Faculty of Medicine and Biomedical Sciences, University of Yaoundé I, Yaoundé, Cameroon; ^7^ Department of Surgery, Presbyterian Church of East Africa (P.C.E.A) Chogoria Hospital, Chogoria, Kenya; ^8^ Division of Urology, Department of Surgery, University of Ilorin Teaching Hospital, Ilorin, Kwara, Nigeria; ^9^ Department of Surgery, John F Kennedy Medical Centre, Monrovia, Liberia; ^10^ Urology Unit, University Hospital Centre Ibn Rochd - Casablanca, Morocco; ^11^ Surgery Department, Kati University Hospital, Kati, Mali; ^12^ Department of Urology, Kilimanjaro Christian Medical University College, Moshi, Tanzania; ^13^ Department of Surgery, Faculty of Human Health Sciences, University of N’djamena, N’djamena, Chad; ^14^ Faculté de Médecine et de Pharmacie, Université Hassan II de Casablanca, Casablanca, Morocco; ^15^ Department of Clinical Sciences, Faculty of Health Sciences, University of Bamenda, Bamenda, Cameroon; ^16^ Département de Chirurgie et Spécialités Chirurgicales. Faculté de Médecine, de Pharmacie et d’Ondo-stomatologie, Université Cheikh Anta Diop, Dakar, Senegal; ^17^ Division of Urology, Department of Surgery, College of Medicine, University of Ibadan, Ibadan, Nigeria

**Keywords:** prostate, prostate-specific antigen, urologist, prostate cancer, Africa

## Abstract

**Introduction:**

In Africa, prostate cancer poses significant diagnostic and treatment challenges due to limited access to diagnostic tools and healthcare resources. This survey aimed to assess current prostate biopsy practices, patient classification methods, and available therapeutic means among African urologists to propose strategies for improved screening, diagnosis, and management.

**Methods:**

A web-based self-administered questionnaire was distributed to urologists in 58 African centres, focusing on biopsy practices, cancer diagnosis, management, and treatment protocols. After pretesting and validation, data collection spanned six weeks, followed by duplicate elimination and arithmetical analysis, with results expressed in absolute, mean, or percentage values.

**Results:**

Feedback was received from 58 centres across diverse African regions, including Central, Southern, West, East Africa, and Madagascar, comprising general, private, and university hospitals. Prostate cancer emerged as the most frequent urological cancer in all regions studied. The assay for prostate-specific antigen (PSA) was available in nearly all centres. Biopsy techniques varied, with ultrasound-guided biopsies being the most common (30 centres), followed by digital-guided (20 centres) and MRI-guided biopsies (5 centres). One centre lacked the expertise to perform biopsies. Radiological workup availability was high, with CT available in 56 centres, MRI in 54, PET scans in 14, and scintigraphy in 29. Treatment capabilities varied, with 53.4% of centres able to perform radical prostatectomies, 86.2% offering radiotherapy, and 94.8% providing medical castration. Among the centres, 56 performed fewer than 5 radical prostatectomies per month, while only 2 centres performed between 5 and 10 per month.

**Conclusion:**

It is important to standardise prostate cancer diagnosis and treatment protocols across Africa while improving access to advanced diagnostic technologies and treatment facilities. Implementing these changes could enhance early detection, improve treatment outcomes, and reduce the burden of prostate cancer in Africa.

## 1 Introduction

Prostate cancer is the second most common cancer and the fifth leading cause of cancer death in men in 2020, according to global cancer statistics. Its incidence varies from country to country (1). This variation can even exist within a single country. In the United States, it has been shown that the incidence of prostate cancer is higher in African-Americans than in Americans of other origins (2). Prostate cancer is also the leading cause of cancer death among men in 48 countries, including many in sub-Saharan Africa (1). In many countries or geographical areas, population-based epidemiological data are available (3–5). In Africa, only a few countries have population-based cancer registries, and these are not well equipped or regularly updated (6, 7).

The diagnosis of prostate cancer is traditionally based on a combination of clinical, biological and histological data. Clinically, diagnosis is made by digital rectal examination, which assesses the shape, regularity and consistency of the prostate (8). Any abnormality of the prostate on digital rectal examination is an indication for prostate biopsy (9). Biologically, prostate cancer is suspected on the basis of elevated plasma levels of prostate-specific antigen. Prostate-specific antigen is a glycoprotein normally expressed by prostate tissue and can also be elevated in men even when there is no prostate cancer involvement (10). In the PSA-based screening program, most patients diagnosed with prostate cancer have a normal digital rectal examination (8). Thus, for diagnostic confirmation of prostate cancer, a prostate biopsy is mandatory. The standard technique is ultrasound-guided needle biopsy of the prostate, taking 12 cores per extended sextant (8).In selected patients, magnetic resonance imaging may be required for targeted biopsy (11). MRI-ultrasound fusion biopsy, especially in those patients with previous negative prostate biopsy but with persistent clinical signs and symptoms of prostate cancer. Biopsy protocols covering indications, preparation, procedure and management of complications are well defined by various national and international urology societies (12, 13). However, there are still wide differences in prostate biopsy practice patterns, according to a survey of 51 Nigerian urologists, which revealed variation in practice. The authors of this publication concluded that practice standardisation is required to increase consistency in patient treatment (14).

On the basis of a range of clinical, biological and histological data, a classification system has been proposed, stratifying patients into groups and guiding the performance of an extensive workup (thoraco-abdomino-pelvic CT and bone scan) (15). Depending on D’Amico’s classification and the presence or absence of metastases on imaging, several learned societies have defined recommendations for practice (12, 13).

There has been a reported rise in the cancer burden in sub-Saharan Africa (16). In terms of incidence and mortality, prostate cancer is the most common cancer among men in sub-Saharan Africa (17). In addition to this huge burden, diagnosing and treating prostate cancer is challenging in sub-Saharan Africa. This is due to lack of access to diagnostic tools and limited healthcare resources. PSA testing, magnetic resonance imaging and biopsy are often limited. Treatment options are often limited by a lack of qualified human and material resources (18). Given the above, we have asked 3 questions within the context of sub-Saharan:

1. What is the current practice of urologists regarding prostate biopsy across Africa?
2. How do urologists classify patients to frame a therapeutic plan?
3. What therapeutic means are available, and what are the selection criteria?

To answer these questions, we surveyed urologists practicing in hospitals in various African countries. The aim of this survey is to give a general overview of the current practice, to compare and contrast the various practice protocols and to propose strategies and recommendations for better screening, diagnosis and management of prostate cancer.

## 2 Methodology

The Modern Urology for Africa (MUFA) association is a recently established continental initiative aimed at advancing urological care in Africa. MUFA conducted this qualitative multicentric survey as one of its diversified scopes of research activities. A web-based, self-administered questionnaire targeting Africa’s prostate biopsy and cancer management practices was designed and electronically disseminated to urologists in 45 African urology centres. These were mainly African public health care institutions with a functioning urology unit or a surgery unit with an attending urologist.

The questionnaire contained the following questions specific to prostate cancer:

- Prostate biopsy practice: patient consent, prostate biopsy preparation, monthly biopsies performed on average, biopsy technique and post-biopsy management.
- Prostate cancer diagnosis, staging and imaging modalities.
- Prostate cancer management: MDT meetings, management protocol, therapeutic modalities.
- Prostate cancer treatment: androgen deprivation therapy (medical or surgical), chemotherapy, radiotherapy, and immunotherapy.

After pretesting and validation of the form by the authors, a Google Form was prepared and sent out through email and WhatsApp to African urologists for automatic data collection. The study was launched on February 12, 2024 and lasted for 6 consecutive weeks. Following data collection, a double check was carried out by authors to eliminate duplicates, and an arithmetical analysis was done. Results were expressed as absolute, mean or percentage values. No correlations were performed.

## 3 Results

**Figure 1:**
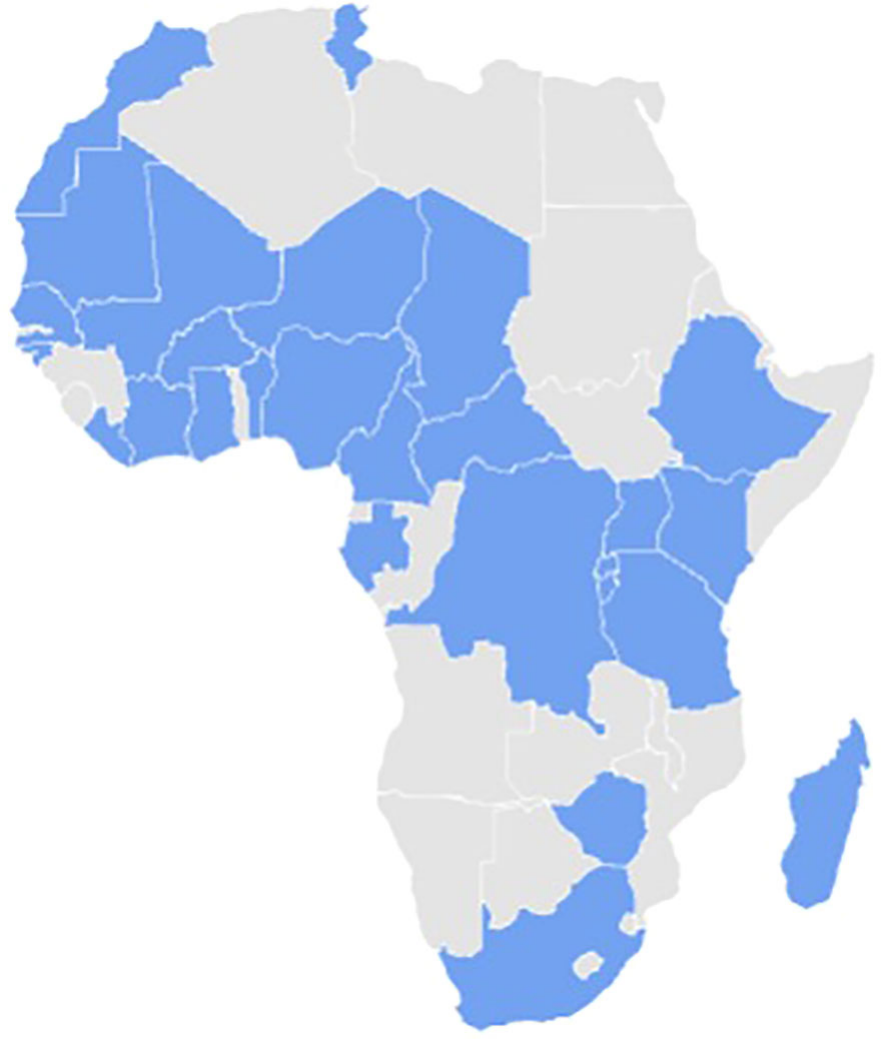
Regions and 28 countries represented in the survey (marked in blue).

We received feedback from 58 centres spanning diverse African regions, such as Central Africa, Southern Africa, West Africa, East Africa, and Madagascar, like indicated in **Figure 1**.

These institutions comprised general community hospitals, academic affiliated hospitals, and university hospitals. **Table 1** displays the breakdown of participating hospitals categorised by their respective African regions.

**Table 1:** Hospitals by African Region participated to the survey.

Geographic area	Centre	Countries
West Africa	Centre Hospitalier National Porto Novo	Bénin
	Centre National Hospitalier Universitaire Hubert Koutoukou Maga (CNHU - HKM)	
	Centre Hospitalier Universitaire Yalgado Ouedraogo	Burkina Faso
	Centre Hospitalier Régional Tenkodogo	
	Hôpital de l'Amitié	
	Centre Hospitalier Universitaire Bogodogo	
	Centre Hospitalier Universitaire de Cocody	Côte d’Ivoire
	Cape Coast Teaching Hospital	Ghana
	Hospital Nacional Simao Mendes	Guinea-Bissau
	John F. Kennedy Medical Centre	Liberia
	Centre Hospitalier Universitaire Kati	Mali
	Hôpital National de Zinder	Niger
	Hôpital national Amirou Boubacar Diallo	Nigeria
	University of Ilorin Teaching Hospital	
	Federal Teaching Hospital Gombe	
	Federal Medical Centre Abuja	
	Usmanu Danfodiyo University Teaching Hospital,	
	Alex Ekwueme Federal University Teaching Hospital	
	Benue State University Teaching Hospital	
	Tetfund Centre of Excellence in Urology and Nephrology	
	Sokoto University of Benin Teaching Hospital	
	University of Maiduguri Teaching Hospital	
	Federal Medical Centre Yenagoa Bayelsa State	
	Centre Hospitalier National Cheikh Ahmadoul Khadim	Sénégal
	Centre Hospitalier National de Pikine	
	Centre Hospitalier Régional de Fatick	
	Hospital Général Idrissa Pouye	
	Hospital Dalal Jamm	
Central Africa	Bertoua Regional Hospital	Cameroon
	General Hospital of Douala	
	Hôpital de District de Bonassama	
	Hôpital Central de Yaoundé	
	Hôpital Laquintinie de Douala	
	Hôpital de L'Amitié Sino-centrafricaine	Central
	Centre Hospitalier Universitaire La Renaissance	Chad
	Centre Hospitalier Universitaire de Référence Nationale	
	Hôpital de l'Amitié Tchad-Chine	
	Centre Hospitalier Universitaire de Brazzaville,	Congo Brazzaville
	Centre Hospitalier Universitaire de Libreville	Gabon
East Africa ** ** ** **	Worabe Comprehensive Specialized Hospital	Ethiopia
	Wachemo University College Nigist Eleni Mohammed General Hospital	
	Hawassa University Comprehensive Specialized Hospital	
	Tenwek Hospital	Kenya
	PCEA Chogoria Hospital	
	Centre Hospitalier Universitaire Joseph Ravoahangy Andrianavalona,	Madagascar
	Kilimanjaro Christian Medical Centre	Tanzania
	Bugando Medical Centre	
	Mbeya Zonal Referral Hospital (MZRH)	
	University Teaching Hospital of Kigali	Rwanda
	Mulago National Referral and Specialised Centre	Uganda
	Mulago National Referral Hospital	
North Africa	Centre Hospitalier Universitaire Ibn Rochd,	Morocco
	Centre Hospitalier Universitaire Hassan II	
	Hôpital Tahar Sfar de Mahdia	Tunisia
	Habib Thameur Hospital	
Southern Africa	Groote Schuur Hospital, United Bulawayo Hospitals	South Africa

### 3.1 Diagnosis

**Figure 2:**
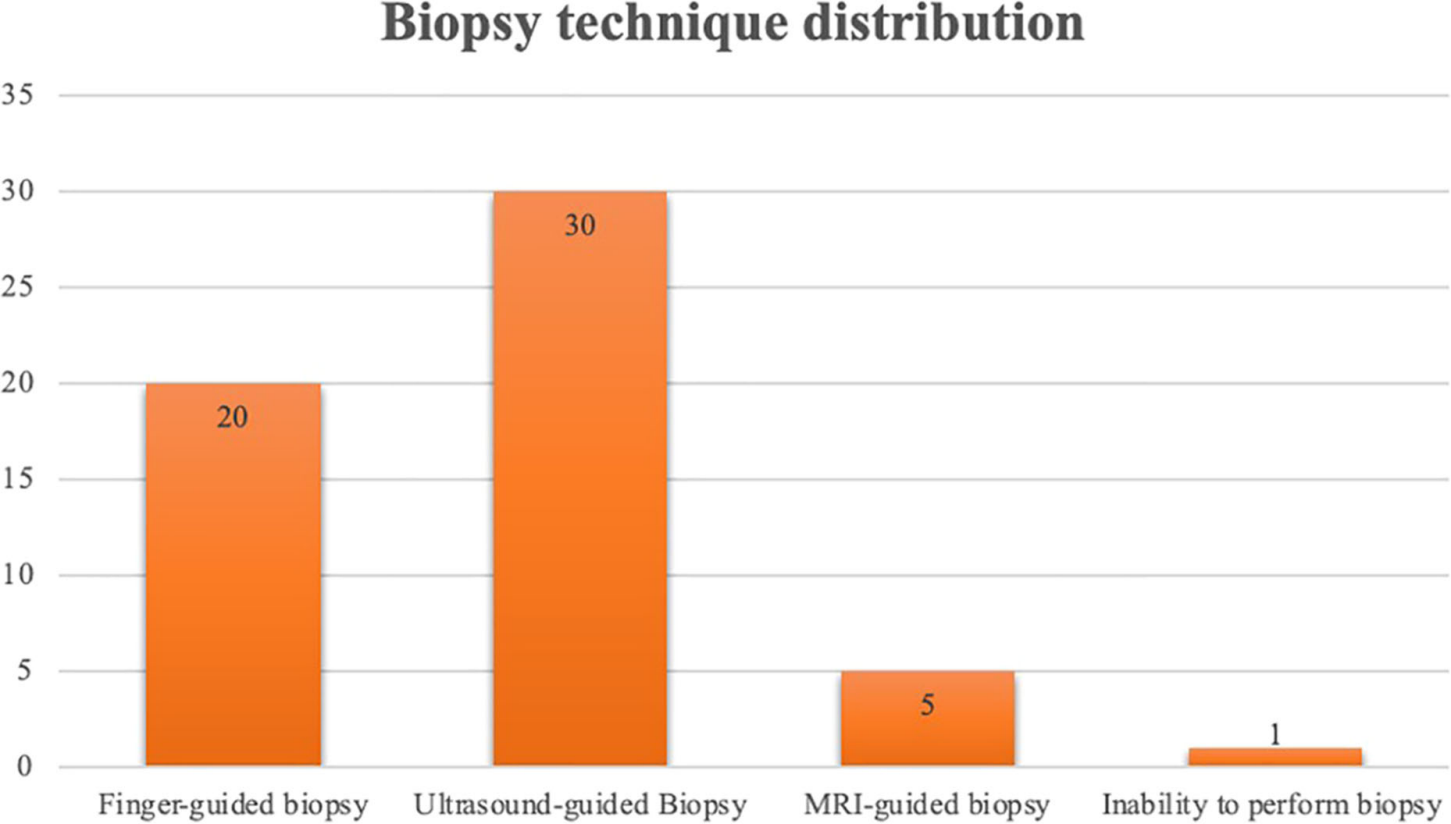
Centres distribution according to Prostate Biopsy Technique.

In all subregions of Africa, i.e., Western, Eastern, Central, and Southern, prostate cancer ranks first in terms of prevalence among the studied urological cancers. Regarding the available screening and diagnostic modalities, the assay for prostate-specific antigen (PSA) is available in almost all the mentioned hospital centres. The majority of centres (n=30, 50%) perform ultrasound-guided biopsies while 20 centres (35%) rely on finger-guided prostate biopsies. Only a smaller number of centres perform MRI-guided biopsies (5 centres). Surprisingly, one centre reported an inability to perform prostate biopsies due to a lack of expertise (**Figure 2**).

**Figure 3:**
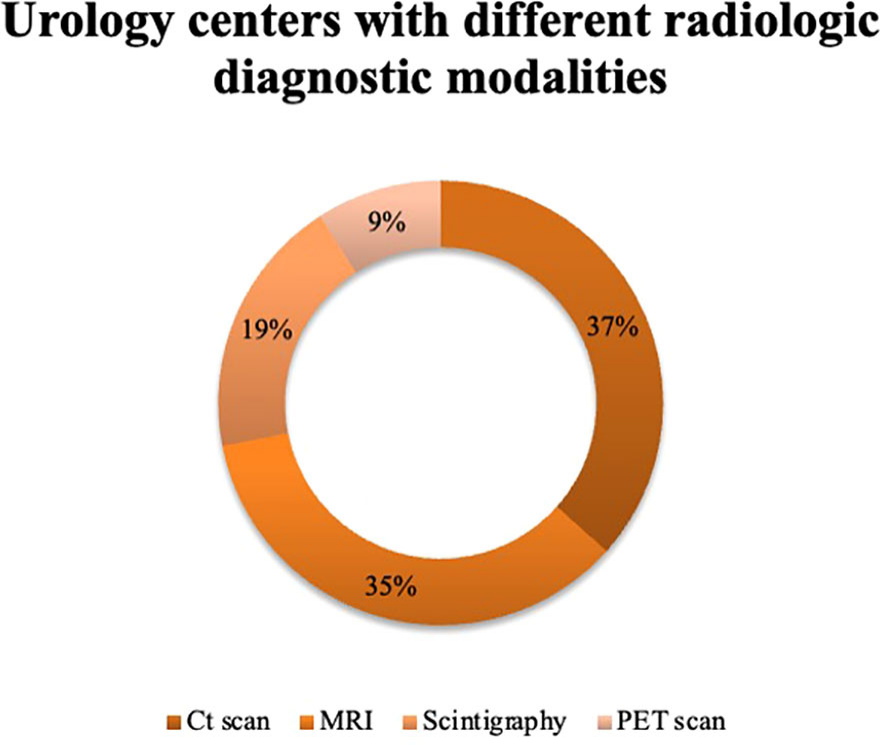
Availability of Radiological Workup in Surveyed African Centres.

The cross-sectional diagnostic imaging modalities available in the various countries were CT scans (56 centres), followed by MRIs (54 centres). For extensive workup, it was possible to do scintigraphy in 29 centres, while only 14 centres could do PET scans. The distribution of imaging modalities is in **Figure 3**.

### 3.2 Treatment

In general, at least 1 therapeutic modality for prostate cancer could be proposed to patients in the centres involved in our survey. The distribution of these modalities varies from one centre to another. The distribution of those various modes of treatment being practiced in the surveyed urology centres is shown in **Table 2**.

**Table 2:** Overview of the availability of Urological Services in Surveyed African Centres.

DIAGNOSTIC AND THERAPEUTIC MODALITIES AVAILABLE	YES	NO
Availability of Radiotherapy centres in the surveyed countries	*50*	*8*
Availability of Genetic analysis in the centre	*19*	*39*
Availability of Immunohistochemistry in your centre	*39*	*19*
Availability of radiological workup in the surveyed centre	*57*	*01*
Availability of Medical castration in the centre	*55*	*3*
Availability of Surgical castration in the centre	*58*	*0*
Ability to perform radical prostatectomy in the country	*46*	*12*
Ability to perform radical prostatectomy in the centre	*31*	*27*

Radical prostatectomy was performed in 53.4% of the centres among the surveyed centres. Out of those 37 centres performing RP, 29 centres do <5 RP per month, while 2 centres do between 5 and 10 RP monthly.

Concerning nonsurgical therapeutic modalities, they were distributed as shown in **Table 3**.

**Table 3:** Availability of non-surgical therapeutic modalities and their distribution among represented hospitals and countries.

Treatment Modality	Centres (n=56)		Countries (n=28)	
	Number (n)	Percentage (%)	Number (n)	Percentage (%)
Chemotherapy	46	82%	27	96
Radiotherapy	18	32%	18	85.7
Brachytherapy	4	7%	4	14.2
Hormonotherapy	47	84%	26	93.1
Cryotherapy	2	3.5%	2	7
Immunotherapy	14	25%	13	46

In regards to radiotherapy, 50 responders (86.2%) reported it was available within their country. We could identify a radiotherapy unit/centre in 18 centres within 18 countries. Mainly, external beam therapy was possible; only 4 centres could offer brachytherapy.

Either surgical or chemical hormonal suppression was possible in all centres, except 3 centres where medical castration was not available. So, only 94.8% of the centres could propose chemical androgen deprivation to patients.

Immunotherapy was offered by 14 centres in 13 countries.

Cryotherapy was only in 2 countries with 1 centre each.

## 4 Discussion

The diagnosis and management of prostate cancer in Africa is of growing concern to the urology community. Although prostate cancer is one of the most common cancers in men, diagnostic and therapeutic practices vary considerably across the continent, influenced by economic, cultural and infrastructural factors. This survey of urologists in various African hospitals provides an overview of current approaches to screening, diagnosis and treatment. By comparing these practices with international standards, this discussion aims to identify specific challenges encountered in Africa, regional variations in management approaches, and opportunities for improving patient outcomes.

### 4.1 Diagnosis of prostate cancer

In western practice, digital rectal exam (DRE) is often performed during screening of prostate cancer and is used as a supporting but not definitive tool to detect prostate cancer. Its capabilities are limited due to its questionable reliability, reduced sensitivity, and inability to explore the entire surface of the prostate, especially when dealing with small tumours that have not yet developed to the point of reaching the prostatic capsule (19). The prostate-specific antigen (PSA) blood test is also another clinical evidence that is used to assess suspicion of prostate cancer (20). In high-income countries, the use of technologies such as MRI is beginning to have a place in prostate cancer screening and surveillance (21). In Africa, DRE and PSA are almost always used for screening and diagnosing PCA. Determining PSA levels depends on the availability of a biomedical analysis laboratory. The results of this survey clearly demonstrated the unavailability of MRI in several centres that responded to the survey.

In 2021, the National Comprehensive Cancer Network (NCCN) developed several harmonised consensus guidelines for sub-Saharan Africa in collaboration with the Africa Cancer Coalition (ACC). One of these guidelines is about early detection of prostate cancer. In this guideline, the NCCN acknowledges that the recommendations on prostate cancer screening methods and intervals are based on expert opinion, and a unanimous consensus has not been reached. Guidelines on when to start and stop screening, screening intervals and when to perform a biopsy are included in the guideline and should be used after the patient has been properly counselled (22). Just recently, the Lancet commission on prostate cancer published an article that draws attention to the increasing number of cases. Among the key points of this publication, it was made clear that late diagnosis of prostate cancer is widespread throughout the world, but particularly in low-income countries. Therefore, they recommended an urgent need to set up earlier diagnosis systems in low- and middle-income countries. For men of African descent, the case for prostate cancer screening lies between the ages of 45 and 70 (21).

### 4.2 Overview of the current guidelines for the diagnosis and screening of prostate cancer

Screening for PCa is an area of frequent debate. Screening practices and guidelines aim to balance the early detection of clinically significant cancers against the risk of potential overdiagnosis and resulting overtreatment of clinically insignificant PCa.

The diagnosis of prostate cancer, like any other cancer type, is highly dependent on histopathological confirmation. The decision to perform a prostate biopsy is made by a urologist based on abnormalities identified during routine screening protocols or after clinical examination of a symptomatic patient (23, 24).

Abnormal findings on DRE, elevation of PSA and a suspicious nodule on mp-MRI are the three most common scenarios that prompt a urologist to perform a prostate biopsy. There is no debate among urologists and oncologists on the need to analyse prostatic tissue samples before diagnosing prostate cancer and planning on the treatment options. However, the question is how these tissue samples are obtained (25). There is certain global variation in practice in this regard, and our study attempted to highlight the practice pattern in Africa.

Until recently, a twelve-core transrectal ultrasound (TRUS)-guided core needle biopsy has been the standard according to the American Urological Association (AUA) and European Association of Urology (EAU) guidelines (26). Recently, a TRUS-guided transperineal biopsy has been recommended by these guidelines mainly to reduce the risk of infection, which is higher in a transrectal biopsy.

Efficient diagnosis and staging of prostate cancer require exhaustive testing techniques specifically designed for the African environment. A prostate biopsy is a crucial diagnostic procedure that is frequently carried out blindly in many African nations. Our results illustrate that biopsy practices vary between regions. According to them, 20 centres were still practicing digital-guided biopsies, and 1 centre in particular could not perform biopsies. On the other hand, switching to image-guided biopsies—ideally using fusion methods that combine MRI and ultrasound—can greatly improve accuracy. We found out that only 5 centres could perform MRI-guided biopsies, but noted a shift toward ultrasound-guided biopsies, which were available in 30 centres. This makes single-use biopsy trocars, biopsy guidance devices, and endorectal ultrasonography probes necessary.

Because image-guided prostate biopsy is a very simple operation to learn, urologists may find it easier to embrace if they participate in short-term training programs in these procedures.

A study reported that twelve procedures are necessary to perform high-quality TRUS-guided prostate biopsies without compromising prostate cancer detection; therefore, they strongly recommended training under direct supervision for a minimum of 12 cases before allowing TRUS-guided biopsy without supervision (27).

In the era of imaging-guided biopsy of the prostate, finger-guided prostate biopsy still has a role in low- to middle-income countries. Concerning finger-guided biopsy and TRUS-biopsy practices in Africa, even if the first one is far from gold-standard, it remains a suitable alternative in resource-limited settings, especially when the prostate is clinically abnormal (28). Perhaps in patients with palpable disease, the addition of finger-guided prostate biopsy of these areas increased the positive predictive value over transrectal ultrasound prostate biopsies alone (29). Also, many countries are still practicing ‘out-of-pocket’ healthcare, and something as simple as a TRUS-guided prostate biopsy may be totally unaffordable. Therefore, in low- to middle-income countries, the cost of a prostate biopsy needs to be considered, as well as the cost to the patient, which may include time off work and travel costs.

We did not distinguish between transrectal and transperineal ultrasound or MRI-guided biopsy technique in our survey; therefore, we could not discuss or compare the advantages of one over the other, including a reduction in infectious complications, improved quality of prostate sampling, and rebiopsy rates, which were reported in a systematic review on biopsy approaches as 15.4% in transrectal vs. 5.26% in transperineal (30).

Even though we did not evaluate prostate biopsy antibiotic prophylaxis protocols applied by each centre in our survey, following a literature review on the topic, we found a study dedicated to analysing post-biopsy complications in relation to antibiotic prophylactic protocols conducted by Tulone et al. They observed that prophylaxis based on the use of fluoroquinolones appears to be associated with a lower risk of infectious complications following transrectal prostate biopsy, compared to other prophylactic regimens, such as those involving cephalosporins or trimethoprim/sulfamethoxazole (31). In addition, another study on the topic conducted in Nigeria comparing two groups of patients among whom Group 1 patients received a single dose of 1 g of amikacin intravenously 30 minutes prior to the procedure, followed by the immediate administration of 500 mg of oral ciprofloxacin after the transrectal ultrasound-guided prostate biopsy (TRUS-PB), while Group 2 patients were given the same antibiotics, but with a notable difference: ciprofloxacin was initiated one day before the procedure. Both patient groups were subsequently administered ciprofloxacin twice daily for a total duration of five days (32). They found a significant reduction in the incidence and severity of sepsis in the second group. In this study, it was requested that antibiotics be used. We wanted to see clinical habits and compare them with the recommendations of learned societies.

Recommendations on prostate cancer screening and early detection from main international urology societies, including AUA, EAU and NCCN guidelines, are highlighted and summarised in **Table 4** (22, 33).

**Table 4:** A summary of society's recommendations on prostate cancer early detection protocol.

Guideline	Who should be screened? *	Frequency of screening	Method of screening	When to do biopsy	mp-MRI
AUA 2023	· Age 50-69 yrs. · Age 45-69 yrs. for high-risk men**	·Every 2 – 4 yrs.	· Serum PSA is the first line. · DRE can be an adjunct modality	· Elevated PSA† · Abnormal mp-MRI	· Before a repeat biopsy. · Before initial biopsy if available
EAU 2024	· Age > 50^b^ · Age > 45 (high risk men) ** · Age > 40 for BRCA2 mutation	·Every 2 yrs.	· Serum PSA · DRE is equally useful	· Elevated PSA > 3ng/ml · Abnormal DRE	· Before a repeat biopsy. · Before initial biopsy if available
NCCN 2024	· Age 45-75 yrs. · Age 40-75 yrs. For high-risk men**	· Every 2-4 yrs if PSA <1ng/dl · Every 1-2 yrs if PSA <3ng/dl	· Serum PSA · DRE is adjunct	· Elevated PSA >3 ng/dl · Suspicious DRE	· Before prostate biopsy (if available)

^*^AUA and EAU recommend clinicians to engage in shared decision making with men regarding prostate cancer screening so they can make an informed choice and discourage ordering a PSA test without informing the patient upfront.

^**^High risk men are those with black ancestry, germline mutations (*BRCA1, BRCA2*), strong family history of prostate cancer.

^†^The definition of elevated PSA is age dependent. The threshold is as low as 2.6 ng/ml for younger men.

^b^The EAU recommends to avoid/stop prostate cancer screening in men with < 15 yrs. Life expectancy.

The commonly used prostate-specific antigen (PSA) screening is not widely accessible or affordable in this region. To address this, alternative screening methods like risk assessment approaches and cost-effective PSA tests are being explored to target high-risk individuals and improve screening access (18).

### 4.3 How these guidelines are implemented in practice in various regions of Africa

Clinical practice guidelines (CPGs) serve as frameworks to unify diagnostic criteria and guide clinical decision-making and to promote the highest standards of urological clinical care through education, research and the formation of health care policy. In Africa, the limited resources may preclude urologists from strictly adhering to international guidelines and recommendations. In addition, screening practices and guidelines for PCa and their availability vary across Africa (24). So, some countries have developed their own national guidelines. The American Urological Association (AUA), the European Urological Association (EAU), and the National Comprehensive Cancer Network (NCCN) have standardised guidelines for the management of prostate cancer. From those guidelines, African management of prostate cancer is extrapolated. As an example in South Africa, local practitioners were commissioned to revise international CPGs with a view to applicability to South African clinical practice (34). Prostate cancer screening consists of systematic screening for the disease in an asymptomatic population. This early screening aims at early detection of these diseases and risk stratification; unfortunately, the major challenge experienced in low- and lower-middle-income countries (LLMICs) is the late presentation of PC patients in health facilities due partly to lack of screening, highlighting the need for accessible and affordable early detection programs (35). Despite the fact that mass screening is not recommended compared to individualised informed screening due to the risk of overdiagnosis and overtreatment, many African countries, especially in SSA, still practice population-based screening, often initiated by local hospitals through public health campaigns. For example, in a community-based screening program in Nigeria, 10% of men 50 years of age and over had a PSA over 4 mg/ml, and most had a PSA greater than 10 mg/ml. 8.5% were biopsied, and 1% had histologically confirmed prostate cancer; no information was provided about treatments and outcomes (36). South African guidelines recommend that PCa screening (PSA and digital rectal examination [DRE]) should be performed in all men from 45 years onwards in the absence of identifiable risk factors and from 40 years in black men, where there is a family history of PCa and other identifiable risk factors (37).

While international guidelines are shifting toward transperineal prostate biopsy and MRI-fusion biopsy, in our context, TRUS-guided biopsy as well as finger-guided biopsy are still commonly done (14).

Treatment with curative intent is not widely available and is often very costly in SSA. Currently, there is no consensus on the management of prostate cancer in Africa, as most of the treatment options are directly adopted from international guidelines. The availability of treatment modalities, namely, androgen-deprivation therapy, chemotherapy, radical prostatectomy, external beam radiotherapy and brachytherapy, varied widely among the countries. Notwithstanding, several authors have proposed prostate cancer updates depending on the stage and African subregion (18, 24, 38–40).

### 4.4 Challenges or barriers that exist in implementing these guidelines in different regions

Diagnosis of prostate cancer in Sub-Saharan Africa is challenging due to limited access to diagnostic tools and healthcare resources, in addition to a low level of awareness by the community and the myths and misconceptions associated with it. The misconceptions include denial of conjugal rights if diagnosed with PCa, and one of the prevention methods is having multiple sexual partners, which predisposes for sexually transmitted disease and often judgement from the community (41). Clinical evaluation and digital rectal examination are commonly used, but PSA testing, magnetic resonance imaging, and biopsy are often restricted to a few locations. Consequently, many men in this region are diagnosed with advanced-stage cancer (39). The data from our study demonstrate an unequal and insufficient distribution of medical devices in healthcare facilities across Africa necessary for the diagnosis and management of prostate cancer. For example, 22 countries have centres capable of performing a computerised tomography (CT) scan, 21 countries have centres with MRI facilities, 10 respondents’ countries have centres equipped with scintigraphy, and only 5 countries have centres capable of performing a positron emission tomography (PET) scan. This aligns with data obtained from the Global Health Observatory in 2014 (42), which showed the limited availability of cancer care equipment in Africa like mammography units, radiotherapy units, CT scanners, gamma camera units, MRI machines, and PET scanners. Radiotherapy centres are also scarce, especially in sub-Saharan Africa. The availability of multimodal PCa treatment in various African countries is key to effectively managing this disease and adhering to standard recommendations. Countries such as Burundi, Guinea-Bissau, Liberia, and the Central African Republic, according to this study finding, do not have radiotherapy centres, forcing eligible patients to seek treatment in neighbouring countries with associated economic costs. In Eswatini, the costs associated with prostate cancer (PCa) management are overwhelming. Due to the absence of radiation therapy in the country, the government must refer PCa patients to private hospitals in South Africa. While surgical procedures like transurethral resection can be performed locally, most patients also seek chemotherapy treatment in South Africa. The total annual cost of PCa to Eswatini is estimated at $6 million, including direct expenses for treatments and non-medical costs like transportation and lodging, which impose a significant financial burden on patients (43).

Urologists in Africa may encounter difficulties adhering to international recommendations due to these resource and knowledge limitations. In our study, it was observed that not all urological centres perform radical prostatectomy due to a lack of technical expertise and adequate equipment. Furthermore, among those centres that do perform this procedure, the average monthly number of prostatectomies conducted by centres indicating affirmative responses was approximately 5.33 cases per year. Our findings also highlight there are centres that do not offer radical prostatectomy services, which significantly impacts both the quality of patient care and training of urologists.

The difficulty for African urological centres to accurately diagnose different stages of prostate cancer, due to limitations in existing diagnostic tests, can result in either underdiagnosis or overtreatment, posing a barrier to the implementation of the best international recommendations. Furthermore, the lack of attention given to the severity of prostate cancer in men compared to other types of cancer in women, such as breast and cervical cancer, can lead to a lack of attention and resources for the screening and early management of prostate cancer. The fact that many cases of prostate cancer in Africa remain undiagnosed and untreated reflects systemic barriers to accessing healthcare and standard treatment. In particular, the centralisation of oncology facilities (including investigations, radiotherapy, and chemotherapy) in urban areas and the shortage of specialised urologists and oncologists make access to care even more difficult for patients in rural areas. Public health expenditures are only $8 per capita, significantly below the recommended $34. Most healthcare costs are paid out-of-pocket, comprising around 70% of total health spending (44). The National Health Insurance Scheme (NHIS) mainly covers civil servants, leaving a large portion of the population uninsured. Endemic poverty exacerbates the situation, making healthcare unaffordable for many patients and leading to delays in seeking medical care (45). These systemic and organisational challenges can affect the quality of urological practices in Africa and explain why they may not always meet international standards. Aligning with international standards for prostate cancer management requires an upgrade of the technical infrastructure in African hospitals for prostate cancer diagnosis, improving the accessibility of these technical platforms to all patients, as well as strengthening training programs for healthcare professionals, including urologists, oncologists, and radiotherapists, to ensure they have the skills and expertise needed to provide quality care to prostate cancer patients.

### 4.5 Limited access to diagnostic and treatment resources and up-to-date training for SSA Urologists

With the recent advancement in the standardised reporting of prostatic MRI and the wide availability of it in higher-income countries, it has been used for the detection, localisation, and characterisation of clinically significant prostate cancers (46). This recent development of using MRI as a screening tool in the detection of prostate cancer has aided urologists and oncologists in performing targeted biopsies and also avoiding unnecessary biopsies. The impact of these targeted biopsies is very high, especially in patients with prior negative biopsy results and still suspicious DRE and PSA results. In this survey, 46.7% (n=21) centres only have access to MRI, which will affect the quality of service provided, resulting in underdiagnosis by missing the cancer on biopsy or increased performance of biopsies.

Radical prostatectomy has undergone a very rapid change from an open approach to a laparoscopic and robotic approach. From initial reports of both laparoscopic and robotic approaches in the beginning of 2000, it has gained wide popularity (47, 48). Now, minimally invasive radical prostatectomy, specifically robotic, is the standard. 91.5% of radical prostatectomies and more than 84% of prostatectomies in England and the USA, respectively, were done robotically (49). In Sub-Saharan Africa, few centres are able to perform radical prostatectomies, and usually it is an open approach. The eligibility of patients for this curative approach is also dependent on early presentation, proper staging, and the availability of skilled urologists who can offer the surgery.

South Africa is the first African country to perform robotic radical prostatectomy and also has many robotic surgery centres (50). The price of acquiring robots and the running cost per procedure have made it unthinkable for most African centres. In addition to its expensiveness, the unavailability of training centres in Africa and the difficulty of securing on-hand training in high-income countries make even the disparity in radical prostatectomy an ongoing challenge. The Vattikuti Foundation has played a significant role in training more than 50 fellows in robotic surgery in India (51). Africa also needs collaboration from this type of foundation and international stakeholders to train and equip the urologists with the necessary skills.

### 4.6 The need for strengthening the technical skills

It is important for urologists to remain up-to-date regarding research and clinical guidelines within their specialty. Prostate cancer rarely involves a single treatment; patient care can be as diverse as the disease itself. Its management often requires a multispecialty team dedicated to managing the diagnosis, evaluation, and treatment. This approach has been reported to improve quality-of-life issues for these patients. The availability of primary treatment modalities, namely, radical prostatectomy, external beam radiotherapy and brachytherapy, varied widely among the countries in which the urologists practiced based on their referral patterns.

Uro-oncology has emerged as a subspecialty that plays an important role in the daily practice and care of urologic malignancies. Its role is so important in standardising the care and in a continuation of urologic oncology training, especially with updated guidelines, changing treatment modalities, new oncological drugs, developments in advanced technology, and the increasing use of laparoscopic, robotic, and endoscopic techniques. A study showed that 40.54% of the urologists did not find the uro-oncology training in their own country to be sufficient, so there is a need for training after residency to be supported by not only the authorities but also the urologists in the field to strengthen their skills (52). Also, surgery training is essential; this is illustrated by the results of multi-institutional analysis, which showed that only 11% of patients treated by high-volume surgeons experienced a biochemical recurrence at five years, compared to 18% of patients treated by low-volume surgeons (53). Performing radical prostatectomy is a key tool for urologists dealing with prostate cancer, especially for eligible patients. Mastering this technique comes with training and practice. Postoperative morbidity in high-volume centres has a 6% absolute reduction in postoperative complication rates (26% vs. 32%), an 8% absolute reduction in late urinary complications (20% vs. 28%), and a 4% absolute reduction in long-term incontinence (16% vs. 20%), compared to those treated by low-volume surgeons (54). Apart from fellowship, urologists indicated an interest in taking courses designed to educate them on prostate cancer care even after residency. Consequently, they should be encouraged to participate in continuous medical education and workshops, to be members of different societies and groups, to subscribe to journals or newsletters, to attend scientific meetings and programs, and to collaborate with other fields. Guidelines are developed to improve the quality of patient care.

### 4.7 Implications for clinical practice

This study has shown a significant disparity in diagnostic and treatment resources between different centres, not only based on the country but also within the same country. This situation has a considerable practical impact, particularly on the reliability of diagnosis, the quality of disease staging, and especially patient management. Even though many efforts have been made to improve diagnostic means, as highlighted by our study (all centres perform prostate biopsies except one), many patients are diagnosed at a late metastatic stage, mainly due to the absence of early screening or the unavailability of tests. Digital rectal examination and PSA testing are available in almost all centres. In a review, Jalloh et al. reported that the unavailability of certain equipment, the lack of expertise among human resources, and the high cost of exams and treatments result in each centre operating according to the available means (18). Similarly, it was reported by Tanzanian authors that there is a significant disparity in the prices of diagnostics and treatments between different centres in the country, and these prices remain relatively high compared to the patients’ means (55). It would be beneficial to establish or encourage collaboration between different centres within the same country and the sub-region, which would increase the level of detection and patient management.

### 4.8 Study limitation

There was no systematic sampling for selecting the hospitals involved in this study, and therefore the possibility of sampling errors exists, and the results may not reflect the overall picture of prostate cancer diagnostic and treatment modalities in Africa. Our sample size and unknown response rate may not represent the population for which the survey was intended.

These may mean our results may be subject to the influence of chance factors. Although there was a mixture of different African country representatives, information about the unrepresented countries may have changed our result significantly. It is also important to recognise that the responding urologist may have limited knowledge about the countries he is practicing in. The questions were not competency-based and did not assess deficiencies of skills or knowledge in PCa. Therefore, it was not possible to determine whether any deficiencies in skills or knowledge were significant enough to affect their response. Furthermore, the possibility of response bias cannot be completely eliminated in this study.

## 5 Conclusion

The diagnosis and management of prostate cancer in Africa face significant challenges due to late diagnosis, inadequate manpower, poor health facilities, and limited treatment options. Our survey highlights disparities in diagnostic capabilities, with some centres lacking essential imaging modalities and biopsy techniques. Treatment options also vary widely, with only a few centres offering comprehensive care, including surgery and advanced therapies. There’s a need to improve early detection and patient outcomes by increasing public awareness, developing screening programs, and investing in novel diagnostic and treatment options. Strengthening technical skills through continuous education and training, enhancing infrastructure, and fostering collaboration among healthcare centres are essential steps toward reducing the burden of prostate cancer in sub-Saharan Africa.

## Data Availability

The original contributions presented in the study are included in the article/supplementary material. Further inquiries can be directed to the corresponding author.
